# MutS Homologues hMSH4 and hMSH5: Genetic Variations, Functions, and Implications in Human Diseases

**DOI:** 10.2174/1389202911314020002

**Published:** 2013-04

**Authors:** Nicole Clark, Xiling Wu, Chengtao Her

**Affiliations:** ¶ STARS Program, College of Veterinary Medicine, Washington State University, Pullman, WA 99164-7520, USA; † School of Molecular Biosciences, College of Veterinary Medicine, Washington State University, Pullman, WA 99164-7520, USA

**Keywords:** DNA damage response, Double-strand break (DSB), DNA mismatch repair (MMR), Homologous recombination (HR), MSH4, MSH5, MutS homologues, Single nucleotide polymorphism (SNP).

## Abstract

The prominence of the human mismatch repair (MMR) pathway is clearly reflected by the causal link between MMR gene mutations and the occurrence of Lynch syndrome (or HNPCC). The MMR family of proteins also carries out a plethora of diverse cellular functions beyond its primary role in MMR and homologous recombination. In fact, members of the MMR family of proteins are being increasingly recognized as critical mediators between DNA damage repair and cell survival. Thus, a better functional understanding of MMR proteins will undoubtedly aid the development of strategies to effectively enhance apoptotic signaling in response to DNA damage induced by anti-cancer therapeutics. Among the five known human MutS homologs, hMSH4 and hMSH5 form a unique heterocomplex. However, the expression profiles of the two genes are not correlated in a number of cell types, suggesting that they may function independently as well. Consistent with this, these two proteins are promiscuous and thought to play distinct roles through interacting with different binding partners. Here, we describe the gene and protein structures of eukaryotic MSH4 and MSH5 with a particular emphasis on their human homologues, and we discuss recent findings of the roles of these two genes in DNA damage response and repair. Finally, we delineate the potential links of single nucleotide polymorphism (SNP) loci of these two genes with several human diseases.

## INTRODUCTION

MutS is one of the proteins involved in DNA mismatch repair (MMR) in E. coli [1]. Like other MMR proteins, MutS homologues (MSH) are highly conserved, and multiple homologues have been identified in eukaryotes. There are seven known eukaryotic MutS homologues, and MSH4 and MSH5 form an exclusive heterocomplex [2, 3]. In contrast to the other MutS homologues that recognize mismatched nucleotides to initiate the repair process, these two proteins in Saccharomyces cerevisiae appear to be involved in meiotic recombination and do not play an apparent role in MMR [4, 5]. In fact, various studies using budding yeast, Caenorhabditis elegans, and mice indicate that MSH4 and MSH5 are required for the development of viable gametes, and the two proteins appear to function in the same meiotic pathways [4-10]. Consistent with these observations, the mammalian MSH4 and MSH5 genes are abundantly expressed in the meiotic tissues, with relatively high levels of mRNA in the testis [8, 11-16]. Although the mechanistic basis underlying their function in meiosis is not decisively defined, the purified recombinant hMSH4-hMSH5 protein complex has been demonstrated to interact with recombination intermediate structures including the Holliday junction [17]. Collectively, these studies suggest a role for these two proteins in the process of recombinational DSB repair during meiosis.

In non-meiotic tissues and cell lines, low levels of MSH4 and MSH5 expression are readily detectable. However, the expression profiles of these two genes are different [11-16], suggesting that they may not only function as a heterocomplex but also act independently and possibly play diverse roles via interaction with different protein partners. In fact, identification of the proteins that interact with MSH4, MSH5, or the MSH4-MSH5 complex facilitates elucidation of the roles played by MSH4 and/or MSH5 in various functional processes. Recent evidence supports the view that hMSH4 and hMSH5 act in DNA damage response [18-20], DSB repair [19, 20], and immunoglobulin diversity [21]. In addition, emerging studies have linked hMSH4 and hMSH5 SNP loci with a variety of human diseases, including neoplasia, immune diseases, and reproductive disorders. This further indicates that hMSH4 and hMSH5 play diverse roles in both meiotic and mitotic cells.

In this review, we briefly describe the gene structures and polymorphisms of human hMSH4 and hMSH5, examine their binding partners, discuss the potential roles of these two proteins in non-meiotic processes such as DNA damage response and repair, and summarize the linkage studies of their SNPs with human diseases.

### Structures, Polymorphisms, and Interacting Partners

Homologues of the bacterial MMR proteins MutS and MutL have been identified in many eukaryotic species, including human [22]. All eukaryotes possess several MutS homologues; in humans, there are five (hMSH2-6). Many of these proteins seem to function similarly to their bacterial counterparts, playing crucial roles in MMR [22, 23]. hMSH4 and hMSH5 possess high levels of sequence and structural homology to bacterial MutS, but they have not been implicated in MMR [24]. 

Structurally, hMSH4 is a 936-amino acid (aa) protein (with a predicted molecular mass of 104.8 kDa) encoded by a 2808-bp open reading frame composed of 20 exons that span 116 Kb on chromosome 1p31 [24, 25]. hMSH5 is a 834-amino acid protein (with a predicted molecular mass of 92.9 kDa) encoded by a 2501-bp open reading frame composed of 26 exons that span 25 Kb within the MHC (major histocompatibility complex; also referred to as the human leukocyte antigen, HLA) class III region on chromosome 6p21.3 [11]. Both hMSH4 and hMSH5 contain the conserved sequence motifs characteristic of all MutS homologues. These domains include an ATP-binding domain and a carboxyl terminal helix-turn-helix structural motif [11, 25]. The hMSH4-hMSH5 heterocomplex is formed by the asymmetric interaction of the hMSH4 carboxyl terminus (aa 843-936) with the composite domain composed of both amino terminus (aa 1-109) and carboxyl terminus (aa 731-834) of hMSH5 [16] Fig. (**1**). It is postulated that the hMSH4-hMSH5 heterocomplex forms a sliding clamp structure that stabilizes various recombination intermediate structures during meiosis and DSB repair [17, 24, 26]. 

In humans, hMSH4 and hMSH5 are associated with several coding region non-synonymous SNPs. For hMSH4, these include A60V, A90T, A97T, E162K, I365V, Y589C, and S914N. Of these, only S914N is located in the hMSH5 interaction domain. The hMSH5 variants include P29S, L85F, Y202C, V206F, R351G, L377F, and P786S. Of these, P29S, L85F, and P786S are located in the hMSH4 interaction domains. Mutations in the interaction domains could interfere with proper hMSH4-hMSH5 interaction and thus disrupt the function of both the hMSH4-hMSH5 heterocomplex and other complexes that rely on hMSH4-hMSH5 [24]. Consequently, disruption of proper protein complex formation could have many physiological implications.

The expression patterns of hMSH4 and hMSH5 are unique in different organs. hMSH4 is highly expressed in the testis, while low levels of hMSH4 have also been identified in several tissues throughout body, including the ovary, thymus, colon, pancreas, brain, heart, liver, and placenta [15]. hMSH5 is expressed in virtually all tissues, including meiotic tissues (testis and ovary) as well as non-meiotic tissues such as thymus, skeletal muscle, bone marrow, spinal cord, trachea, and lymph node [11, 12, 16]. These relatively broad expression patterns suggest that hMSH4 and hMSH5 are involved in both mitotic and meiotic processes. The differential expression patterns indicate that hMSH4 and hMSH5 have independent functions in addition to those of the hMSH4-hMSH5 heterocomplex [24]. This hypothesis is supported by studies showing that hMSH4 and hMSH5 have other binding partners Fig. (**2**). For example, hMSH4 interacts with hMLH1 [27] and its binding partner hMLH3 [28], both of which are MutL homologues involved in MMR [27-30]. hMSH4 also interacts with Von Hippel Lindau tumor suppressor-binding protein 1 (VBP1), an interaction which competes with hMSH5 binding [15]. hMSH5 interacts with non-receptor tyrosine kinase c-Abl, an interaction by which c-Abl phosphorylates hMSH5 in response to DNA damage [18, 20]. The amino terminus (aa 1-109) of hMSH5 interacts with the c-Abl SH3 domain, and the hMSH5 P29S variant causes increased c-Abl phosphorylation activity [18]. The hMSH4-hMSH5 heterocomplex interacts with additional proteins; for example, hMSH4-hMSH5 interaction causes the subsequent recruitment of G-protein pathway suppressor 2 (GPS2) [31], an intracellular signaling protein that may be involved in DNA damage response and homologous recombination (HR) [24]. The dynamic interplay among hMSH4, hMSH5, and their interacting partners enables these proteins to exert diverse cellular functions.

Several alternative transcripts are known to result in variant polypeptides for both hMSH4 and hMSH5. One prominent hMSH4 variant is hMSH4sv (splicing variant), which results from exon 19 skipping [15]. hMSH4sv is an 850-amino acid protein, of which the last 7 amino acids are frame-shifted. As a result, hMSH4sv lacks the conserved carboxyl terminal helix-turn-helix motif in its hMSH5-binding domain and thus cannot interact with hMSH5. However, hMSH4sv maintains its ability to interact with another binding partner, VBP1. Expression of hMSH4sv is ~50% of that of hMSH4 in many tissues [15]. In addition to hMSH4 and hMSH4sv, the hMSH4 gene also encodes other variants – such as ΔhMSH4, which results from exon 6 skipping – that may serve different functional purposes [24]. 

Like hMSH4, hMSH5 has a common variant, hMSH5sv (also referred to as hMSH5a). The retention of the last 51 bp of intron 6 results in an in-frame insertion between codons 179 and 180, and thus the final hMSH5 protein contains a 17-amino acid insertion [16]. However, hMSH5sv displays equivalent ability to bind to hMSH4 [24]. Expression of hMSH5sv transcripts is lower than that of hMSH5 in brain, heart, and skeletal muscle tissue but is higher in breast and lung carcinoma tissues, suggesting that hMSH5sv may be up-regulated in breast and lung cancer cells [16]. Other alternative transcripts of hMSH5 include hMSH5b (contains one extra amino acid between codons 654 and 655 due to retention of the last 3 bp of intron 20), hMSH5c (commonly referred to as the wildtype hMSH5), and hMSH5d (contains the aforementioned 17- and 1-amino acid insertions as well as a 30-amino acid deletion between codons 744-773). Further experimentation is necessary to determine the functional uniqueness and significance of these structural variants [24].

### hMSH4 and hMSH5 in DNA Damage Response and Repair

The well-defined function of MMR proteins is to repair base-pairing errors and insertion or deletion loops (IDLs) arising from DNA replication or recombination. Defects in MMR genes lead to genome-wide mutations and microsatellite instability. Mutations in several MMR genes, particularly those in hMSH2 and hMLH1, cause Lynch syndrome (hereditary nonpolyposis colorectal cancer, HNPCC) and increase the risk of development of a wide variety of sporadic cancers in humans.

While the MMR process in eukaryotes is much more complex than that in prokaryotes, the eukaryote MutS homologues generally function similarly to the bacterial MutS in recognizing DNA base pairing errors. MutSα, a heterodimer of MSH2 and MSH6, binds to single base-base mismatches or small IDL; MutSβ, a heterodimer of MSH2 and MSH3, recognizes larger IDL. The DNA-error-bound MutS heterodimer then recruits a MutL heterodimer, primarily MutLα (MLH1-PMS2), and triggers downstream steps of MMR [32].

In addition to DNA repair, the MMR proteins are believed to be involved in signaling various types of DNA damages. As components of the BRCA-1 associated genome surveillance complex, MMR proteins can interact with proteins that participate in detection of DNA damage and/or control of cell cycle checkpoints. The involvement of MMR proteins in DNA damage response is likely through the coupling of DNA damage detection and cell cycle control, which can be accomplished by either direct DNA lesion recognition or the activation of repeated futile MMR that leads to more severe DNA lesions or replication fork arrest [33, 34]. The signaling roles of MMR proteins have been mainly suggested by functional assessment. For instance, some MSH6 missense mutations cause loss of MMR capability while retaining the ability to mediate apoptotic response to DNA damaging agents [35]. In another study, using human embryonic kidney 293T cells, hMLH1 is found to be essential for both MMR and cellular response to DNA damage induced by the methylating agent N-methyl-N’-nitro-N-nitrosoguanidine (MNNG) [36]. Although only low levels of hMLH1 expression are necessary for functional MMR, a full complement of hMLH1 is required to restore G2(M) cell cycle arrest in response to MNNG [36]. This indicates that MMR and cell cycle signaling are two independent responses of MMR proteins to DNA damages. Deficiency in MMR proteins leads to the loss of MMR and/or proper cell cycle signaling, a mechanism that is postulated to contribute to chemotherapeutic drug resistance or DNA damage tolerance [32, 37].

hMSH4 and hMSH5 have not been experimentally implicated in the MMR process [4, 5, 17, 26], possibly due to differences in amino acid residues compared to other MutS homologues [4]. However, hMSH4 and hMSH5 do have a role in meiosis – particularly recombination for crossing over and gene conversion [4, 5] – and possibly also in mitotic DNA damage response and repair. In fact, many of the proteins involved in meiotic recombination are also important players in mitotic DSB repair. BRCA2, RAD51, the MRE11 complex, and ATM, for instance, are all involved in both meiotic recombination and mitotic DSB repair [38-42]. Meiotic recombination is triggered by programmed DSBs that are generated by meiotic-specific type II topoisomerase SPO11 and completed with repair of DSBs by HR [43]. The involvement of MSH5 and MSH4 in meiotic recombination suggests a potential role of these two proteins in mitotic DSB repair. The most lethal form of DNA lesion, DSBs may arise from replication fork collapse, exposure to DNA damaging agents, or cellular processes requiring programmed DSBs, such as meiotic recombination in germ cells or class switch recombination (CSR) and V(D)J recombination in lymphocytes [44]. In response to DSBs, cells undergo cycle arrest, then either repair the lesions and resume the cell cycle or enter the path of apoptosis, depending on the extent of damage. Cells lacking proper cell cycle checkpoints, apoptotic response, or damage repair are often at a higher risk of malignant transformation. The repair of DSBs generally requires either non-homologous end joining (NHEJ) or HR pathways. While NHEJ is a rapid means utilized by somatic cells to repair DSBs, it is error-prone and can result in alteration in DNA sequences for non-compatible breaks [45, 46]. On the other hand, HR is an error-free repair pathway that occurs mostly at S and G2 phases and utilizes a homologous template provided by either a sister chromatid or the homologous chromosome [47]. Cells missing essential HR proteins often experience chromosome instability and exhibit increased sensitivities to a variety of DNA damage agents, such as IR, cisplatin, and gemcitabine. However, HR-deficient cells may also display DNA damage tolerance and resistance to killing by DNA-damaging agents [48-51].

Supported by protein interactions with hRAD51, c-Abl, hMRE11, and Holliday junction-recognizing protein (HJURP) [18, 24, 52] Fig. (**2**), hMSH4 and hMSH5 are likely to play functional roles in DSB response and repair by preferentially binding to the core of Holliday junctions [17]. Proper DNA damage response and repair is critical in regulating cell cycle checkpoints to prevent cancer development, and dysregulation of proteins like HJURP has been shown to cause chromosome instability at mitotic checkpoints, leading to breast cancer and resistance to radiotherapy [53, 54]. Similarly, recent studies have indicated that hMSH5 promotes apoptosis after IR and de-sensitizes cells to clinical doses of cisplatin [19, 20]. In addition, the interaction between hMSH5 and c-Abl is suggested to play a role in provoking apoptosis particularly after a high dose of IR. While the expression of endogenous hMSH5 is normally maintained at a low level in unperturbed cells, it undergoes IR dose- and time-dependent induction. This induction appears to rely on hMSH5-c-Abl interaction and c-Abl kinase activity. Further, the induced hMSH5 is phosphorylated by c-Abl and enhances c-Abl autophosphorylation. The elevated c-Abl activation stabilizes and activates downstream factor p73 that triggers apoptotic response [19]. In response to cisplatin treatment, the expression of hMSH5 increases and phosphorylation at Tyr742 of hMSH5 arises, apparently by c-Abl kinase activity. In addition, the association of hMSH5 with chromatin significantly elevates, and DNA lesion repair – mostly by HR [55, 56] – is enhanced in human cells. Since silencing hMSH5 expression or replacing hMSH5 with hMSH5 Y742F (a phosphorylation-resistant mutant) sensitizes cells to cisplatin, it appears that the induction of hMSH5 expression and tyrosine phosphorylation of hMSH5 are critical early events for repair of cisplatin-induced DNA lesions [20]. Collectively, these studies suggest that hMSH5, and perhaps hMSH4 as well, play roles not only in DNA damage repair but also in DSB-triggered apoptotic response. 

### Implications in Human Diseases

#### Neoplasia

Neoplasia, or uncontrolled cell growth, can result in malignant (cancerous) or benign tumorigenesis. In general, cancer is a genetically heterogeneous disease that can be caused by the interplay of several mutations in genes responsible for maintaining genomic stability. In various studies, mutations in hMSH4 and hMSH5 have been associated with neoplasia incidence (Table **1**). 

For example, the interplay between variants of hMSH4 and hMLH3 (hMSH4 A97T and hMLH3 L844P) has been shown to be associated with an increased risk for breast cancer in a Caucasian Portuguese population [57]. The structural and functional alteration in hMSH4 A97T and hMLH3 L844P may change their protein interaction properties and thereby affect mitotic recombination in mammary gland cells, leading to increased breast cancer susceptibility. In another study, exposure to elevated levels of estrogen – a risk factor for the development of breast cancer – is shown to result in decreased expression of DNA repair genes including hMSH4 and those involved in MMR in breast cancer cells [58]. Thus, one mechanism whereby estrogen causes breast cancer may be through inhibiting MMR-mediated apoptotic response. This implies that the presence of functional hMSH4 and other MMR-related proteins may be necessary for genomic stability and normal cellular growth, and mutations leading to dysfunctional hMSH4 may be involved in oncogenesis.

The interaction of hMSH4 with VBP1 also suggests a potential link between hMSH4 and Von Hippel Lindau (VHL) Syndrome, a familial syndrome characterized by neoplasms of the retina, kidney, and central nervous system. This syndrome arises from germ-line inactivation of the VHL gene on chromosome 3p25-26 [59]. The VHL protein is shown to bind to VBP1, which is also a binding partner of hMSH4 and hMSH4sv [15] Fig. (**2**). This VBP1-hMSH4 interaction may have an effect on proper chromosome positioning during meiotic chromosome segregation while regulating protein stability [59]. In addition, VBP1 has been shown to bind to Hepatitis B virus X protein (HBx) [60]. Hepatitis B virus is one of the main causes of hepatitis, cirrhosis, and hepatocellular carcinoma. The interaction between HBx and VBP1 synergistically increases cellular proliferation and tumorigenesis [60]. Moreover, hypomethylation of the VBP1 gene has been associated with uterine leiomyoma – the most common benign tumor in women [61]. Proper expression and protein interactions of VBP1 with its binding partners—including VHL, hMSH4, and HBx—are critical for maintaining genomic stability and regulating cell division and proliferation. It is possible that these binding partners exist jointly in protein complexes and that mutation in one binding partner may interfere with the function of the other proteins. Therefore, it is plausible that hMSH4 alterations may contribute to VBP1-related dysfunctions. Additional hints for the potential role of hMSH4 in normal cellular growth has been derived from studies of multiple myeloma, a B-cell malignancy. Loss of chromosome 1p31-32 (a cytoband containing the hMSH4 gene locus) is associated with shorter survival of multiple myeloma patients [62]. However, whether loss of hMSH4 function has any significant effect on myeloma prognosis is not known. More study is needed to elucidate the molecular consequences of chromosome 1p31-32 deletions on the development of myeloma.

Consistent with the idea that hMSH4 and hMSH5 may also function independently, hMSH5 has been implicated in the pathogenesis of different types of neoplasms. For example, the gene locus of hMSH5 on chromosome 6p has been strongly associated with increased risk of lung cancer—particularly non-small cell lung cancer—in a Caucasian population [63]. Specifically, risk loci at 6p21.33—i.e. rs3117582 in intron 1 of BAT3 and rs3131379 in intron 10 of hMSH5—are found to be susceptibility markers for lung cancer [63]. Although it is presently unknown whether rs3131379 could affect hMSH5 function, a recent multilevel association analysis confirms that rs3131379 is a risk factor for lung cancer in Caucasian populations [64]. Interestingly, a non-synonymous hMSH5 SNP (i.e. rs2075789; C85T in exon 2 of hMSH5, leading to the P29S substitution) is not significantly associated with lung cancer risk in a Chinese population [65]. This raises the possibility that the effect of hMSH5 may be modulated by different genetic backgrounds, and susceptibility markers may not be suitable for different ethnic populations. 

Even though the common genetic polymorphism rs2075789 is not associated with lung cancer, it does have implications in other diseases. In fact, rs2075789 has been shown to display a higher allele frequency in females with ovarian carcinoma than that of a control population [16]. The protein encoded by rs2075789 shows decreased ability to bind to hMSH4, therefore having the potential to hinder the formation of the functional hMSH4-hMSH5 heterocomplex. Thus, this variation could affect the subcellular distribution and the protein stabilities of hMSH4-hMSH5 [66, 67]. In addition, this variant protein affects the c-Abl-hMSH5 interaction that is involved in DNA damage response [18]. Thus, rs2075789 is a potential causative factor in tumorigenesis, at least for ovarian carcinoma. 

There is a strong correlation among mutations in DNA damage repair genes, genomic instability, and consequent tumorigenesis. For example, exposure to IR is a well-known factor for the development of gliomas – a common form of primary malignant brain tumors [68]. Thus, variations in DNA repair genes are expected to be important factors for conferring glioma susceptibility. In this regard, hMSH5 synonymous SNP rs707938 is found to be one of the risk loci that are associated with glioma in five case-control studies in Euopean populations [69]. Likewise, recurrent deletions of various chromosome regions harboring DNA repair genes (including hMSH5) have been identified in haematodermic neoplasms, a particularly aggressive form of leukemia [70]. Studies of the expression of MutS homologues in a population of European colorectal carcinoma patients demonstrate that tumors generally contain lower expression levels of all MMR genes [71]. This indicates that losing the function of these proteins—either via mutation or reduced expression—may contribute to genomic instability and tumorigenesis. Collectively, these studies provide several lines of evidence correlating cancer development and genomic aberrations with dysfunctions of DNA repair genes like hMSH4 and hMSH5.

#### Immune Diseases

In addition to its association with neoplasia, hMSH5 alteration has been implicated in several immune diseases. It is noteworthy that the hMSH5 gene is located within the HLA class III region [24], and thus alterations in hMSH5 may concomitantly affect immune system function. In addition, the potential role of hMSH5 in immunoglobulin (Ig) CSR may suggest a causal link between hMSH5 SNPs and immune diseases [21] (Table **1**).

Systemic lupus erythematosus (SLE), an autoimmune disease in which production of autoantibodies causes immune system abnormalities and organ damages, is particularly prevalent in women and African Americans [72, 73]. The complex clinical manifestation of SLE implies genetic heterogeneity and possibly epistatic interactions [74]. Several genomic loci have been identified as susceptibility factors for SLE. Among patients with African American, European, and Asian ancestry, hMSH5 rs3131379 has been established as a susceptibility locus for SLE with genome-wide significance [75]. Studies of SLE cohorts of Spanish and Filipino ancestry indicate that hMSH5-related loci are strongly associated with SLE among various susceptibility loci [76]. Further studies are needed to determine whether hMSH5 SNPs have a functional role in the pathogenesis of SLE or simply serve as a genomic marker. 

Other HLA variations mapped to hMSH5 have also been associated with immune-related disorders. For example, Kawasaki disease (KD)—a complex vasculitis disease associated with immunologic and genetic changes and a leading cause of heart disease in children—has been recently linked to a high susceptibility haplotype that harbors hMSH5 rs1150793 [77]. Susceptibility to the autoimmune disorder type 1 diabetes (T1D), which is caused by autoimmune destruction of pancreatic cells, has been correlated with other SNPs in the HLA class III region. In one study, rs707915—a hMSH5 SNP in a block of six markers linked through Linkage disequilibrium—has been identified as the second- strongest T1D susceptibility marker [78]. Similarly, the hMSH5 rs1150793 that is implicated in KD has been also shown to be associated with susceptibility to severe cutaneous adverse reactions (SCAR) in response to treatment with the anti-hyperuricemia medication Allopurinol in a Han Chinese population. Evidence suggests that this severe reaction to Allopurinol is genetically determined; in particular, HLA presentation of Allopurinol metabolites may activate T cells, and the HLA-B*5801 allele seems to be a genetic marker for this reaction. Thus, the synergistic combination of variations in HLA and hMSH5 may be responsible for mediating Allopurinol-SCAR [79]. Overall, there is no apparent connection between loss of hMSH5 function per se and susceptibility to these diseases. However, the fact that susceptibility to some immune diseases is associated hMSH5 SNPs merits further investigation to determine the potential functional connection between hMSH5 and immune disorders.

One possible role for hMSH5 in immune function is Ig CSR, in which an Ig V(D)J DNA segment is recombined with an Ig constant segment. It has been proposed that the hMSH4-hMSH5 heterocomplex contributes to CSR by suppressing alternative microhomology-mediated end-joining and therefore indirectly promoting resolution of DNA breaks via the NHEJ mechanism [21]. However, studies of two different Msh5 mutant mouse lines have not established a unified role for Msh5 in CSR [21, 80, 81], and further analysis is required to delineate the precise role of hMSH5 in the process of CSR. Given the observation that these two Msh5 mutant mouse lines display meiotic chromosome pairing defects to a different extent [8, 9], it is reasonable to speculate that the role of Msh5 might be modified by the differences in their genetic backgrounds. In Caucasian populations, evidence shows that the hMSH5 rs28381349 (L85F)/rs28399984 (P786S) and hMSH5 intron 12 SNP rs3131378 are enriched in patients with IgA deficiency (IgAD) and common variable immune deficiency (CVID) [21]. The hMSH5 variant L85F/P786S compromises the ability of hMSH5 to interact with its binding partner hMSH4, thereby affecting the formation of functional hMSH4-hMSH5 heterocomplex. However, another study of an IgAD cohort of Spanish ancestry indicates that these hMSH5 SNPs (L85F/P786S and rs3131378) are associated with the presence of IgAD, but hMSH5 per se may not be a factor for IgAD predisposition. Alternatively, these loci can be markers for other causative factors or may interact with other HLA alleles conferring the susceptibility to Ig deficiencies [82]. Further research is clearly warranted to establish the potential population-specific roles of hMSH5 in Ig CSR and the effects of hMSH5 polymorphisms in immune diseases.

#### Reproductive Disorders

The mammalian MSH4-MSH5 heterocomplex is known to exert critical roles in the maintenance of chromosomal stability during meiotic recombination [8-10]. The precise interaction between hMSH4 and hMSH5 is essential in supporting the function of this heterocomplex in processing Holliday junctions during homologous recombination [17, 26]. Disruption of this interaction may lead to errors in recombination, which subsequently lead to chromosome nondisjunction. In fact, mutations resulting in loss of proper function of these proteins are shown to cause reproductive malfunctions (Table **1**), and reduction of binding affinity between hMSH4 and hMSH5 is shown to affect gamete formation. 

For example, the hMSH5 SNP rs2075789, which corresponds to the amino acid substitution P29S, alters the hMSH4-binding domain on hMSH5 and reduces the interacting affinity between hMSH4 and hMSH5 [16]. This SNP has been associated with male infertility, specifically azoospermia and oligozoospermia in a Chinese population [83], and this association has been recently confirmed in another study of a Chinese male infertility cohort [84]. The same SNP has also been associated with premature ovarian failure in Caucasian women [85]. It is conceivable that, besides affecting hMSH4-hMSH5 binding, the hMSH5 SNP rs2075789 may also alter the interplay of hMSH5 with other partners, thus exerting a compounding functional impact on meiotic recombination. Of note, Msh4- and Msh5-null mice display a complete meiotic failure that is subsequently associated with profound testicular and ovarian degeneration [9, 10]. Hypomorphic mutations of hMSH4 and hMSH5 that affect protein interactions may also cause aneuploidy, a result of chromosomal nondisjunction during meiosis [86]. Clearly, proper hMSH4 and hMSH5 function is critical in maintaining normal reproductive health.

## SUMMARY AND CONCLUSION

The fact that mutations in hMSH4 and/or hMSH5 result in pathologies as diverse as neoplasia, immune diseases, and reproductive disorders highlights the importance of hMSH4 and hMSH5 in maintaining genomic stability and normal cellular function in tissues and systems throughout the body. SNPs in hMSH4 and hMSH5 that reduce binding affinity and compromise heterocomplex formation can increase the risk of diseases such as cancer, CVID, and infertility. Notably, the same risk loci have been associated with many different types of diseases (Table **1**). This emphasizes the importance of the identification and functional characterization of hMSH4 and hMSH5 low-frequency SNPs/mutations and their associations with diseases.

It is plausible that different combinations of hMSH4 and hMSH5 SNPs may have unique but subtle impacts on protein interactions and thus influence their functions. In particular, the genetic associations between hMSH4 and hMSH5 mutations and human diseases are not simple one-to-one relations. Complex and dynamic gene-gene interactions involving hMSH4, hMSH5, and others are likely involved in pathogenesis of these diseases. The importance of hMSH4 and hMSH5 in DNA damage response and repair pathways—which have implications in the development of several diseases—merits further research to elucidate the molecular mechanisms and protein interactions involved in hMSH4 and hMSH5 function.

## Figures and Tables

**Fig. (1):**
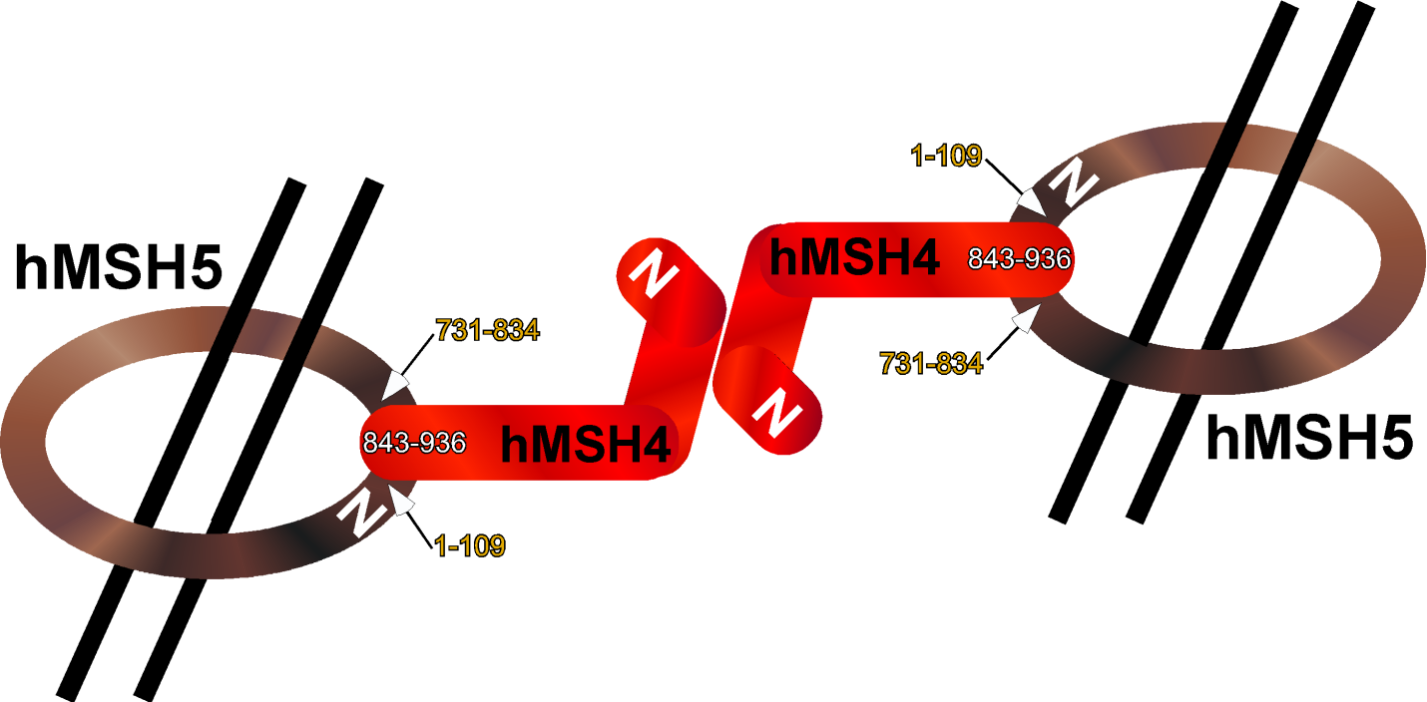
Domains of hMSH4 and hMSH5 proteins that mediate heterocomplex formation. hMSH4 and hMSH5 form a heterocomplex, which is postulated to constitute a sliding clamp structure that stabilizes DNA recombination intermediates. A schematic of the asymmetrical binding between these two proteins is shown. The interaction domains on both proteins are indicated by their corresponding amino acid residue numbers.

**Fig. (2):**
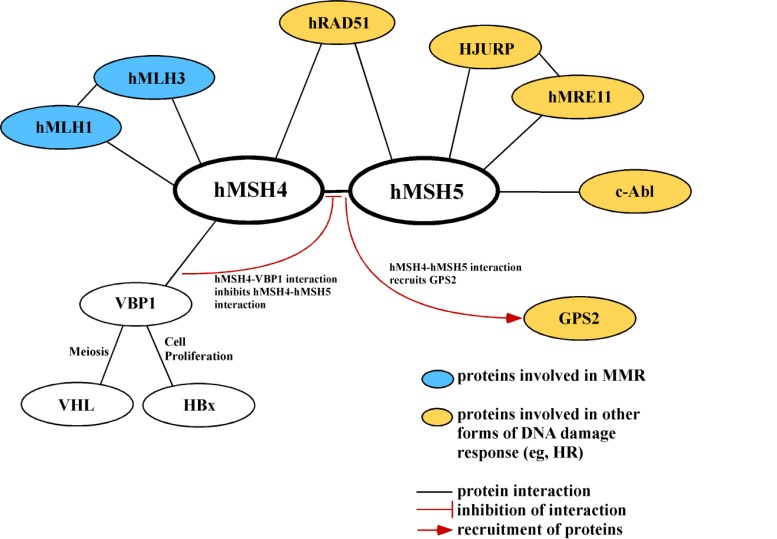
Protein interacting partners of hMSH4 and hMSH5. The interaction between hMSH4 and hMSH5 as well as their protein interaction partners is depicted. Potential cellular functions associated with each of these protein interactions are also indicated (MMR, HR, meiosis, and cell proliferation).

**Table 1.** Implication of hMSH4 and hMSH5 Variants in Human Diseases

Disease Category	Specific Disease	Gene Affected	Risk loci (ID, SNP, and/or amino acid substitution), Possible Effects	Ref.
NEOPLASIA	** Breast Cancer**	hMSH4	rs5745325 (G289A → Ala97Thr) reduction of hMSH4 expression	[57, 58]
	** Von Hippel Lindau Syndrome**	VBP1 (hMSH4 interaction)	Possibly affect VBP1-VHL interaction	[59]
	** Hepatocellular Carcionoma**	VBP1 (hMSH4 interaction)	Possibly affect VBP1-HBx interaction	[60]
	** Uterine Leiomyoma**	VBP1 (hMSH4 interaction)	Possibly affect VBP1-hMSH4 interaction	[61]
	** Myeloma**	hMSH4	Loss of chromosome 1p31.1 (loss of hMSH4)	[62]
	** Lung Cancer**	hMSH5	rs3131379 (intron 10)	[63]
	** Ovarian Cancer**	hMSH5	rs2075789 (C85T → Pro29Ser)	[16]
	** Glioma**	hMSH5	rs707938 (A2148G → Gln716Gln)	[69]
	** Haematodermic Neoplasms**	hMSH5	Deletions in hMSH5 gene	[70]
	** Colorectal Cancer**	hMSH4 and hMSH5	Lower expression of MMR genes	[71]
IMMUNE DISEASES	** Systemic Lupus Erythematosus**	hMSH5	rs3131379 (intron 10)	[74-76]
	** Kawasaki Disease**	hMSH5	rs1150793 (intron 10)	[77]
	** Type 1 Diabetes**	hMSH5	rs707915 (intron 5)	[78]
	** Severe Cutaneous Adverse Reactions (SCAR; response to allopurinol)**	hMSH5	rs1150793 (intron 10)	[79]
	** Selective IgA Deficiency (IgAD) and Common Variable Immune Deficiency (CVID)**	hMSH5	rs28381349 (C253T → Leu85Phe), rs28399984 (C2356T → Pro786Ser), and rs3131378 (intron 12)	[21, 80-82]
REPRODUCTIVE DISORDERS	** Azoospermia/ oligozoospermia**	hMSH5	rs2075789 (C85T → Pro29Ser)	[83, 84]
	** Premature Ovarian Failure**	hMSH5	rs2075789 (C85T → Pro29Ser)	[85]
